# Highway Reconstruction Through Fine-Grained Semantic Segmentation of Mobile Laser Scanning Data

**DOI:** 10.3390/s26010040

**Published:** 2025-12-20

**Authors:** Yuyu Chen, Zhou Yang, Huijing Zhang, Jinhu Wang

**Affiliations:** 1 Aerospace Information Research Institute, Chinese Academy of Sciences, Beijing 100094, China; chenyuyu21@mails.ucas.ac.cn (Y.C.); zhanghuijing@aircas.ac.cn (H.Z.); wangjh@aoe.ac.cn (J.W.); 2 School of Electronic, Electrical and Communication Engineering, University of Chinese Academy of Sciences, Beijing 100049, China; 3 International Research Center of Big Data for Sustainable Development Goals, Beijing 100094, China

**Keywords:** LiDAR, mobile laser scanning, point cloud, semantic segmentation, highway reconstruction

## Abstract

The highway is a crucial component of modern transportation systems, and its efficient management is essential for ensuring safety and facilitating communication. The automatic understanding and reconstruction of highway environments are therefore pivotal for advanced traffic management and intelligent transportation systems. This work introduces a methodology for the fine-grained semantic segmentation and reconstruction of highway environments using dense 3D point cloud data acquired via mobile laser scanning. First, a multi-scale, object-based data augmentation and down-sampling method is introduced to address the issue of training sample imbalance. Subsequently, a deep learning approach utilizing the KPConv convolutional network is proposed to achieve fine-grained semantic segmentation. The segmentation results are then used to reconstruct a 3D model of the highway environment. The methodology is validated on a 32 km stretch of highway, achieving semantic segmentation across 27 categories of environmental features. When evaluated against a manually annotated ground truth, the results exhibit a mean Intersection over Union (mIoU) of 87.27%. These findings demonstrate that the proposed methodology is effective for fine-grained semantic segmentation and instance-level reconstruction of highways in practical scenarios.

## 1. Introduction

Highways are major public roads, typically connecting cities and regions, and are designed to support rapid and efficient transportation. As critical components of modern infrastructure, highways play a vital role in fostering economic development, enhancing regional connectivity, improving safety, and enabling efficient travel and trade. The highway environment mainly comprises multiple lanes, signage, road markings, lighting systems, and guardrails. The automatic extraction of detailed information and the fine-grained reconstruction of highway environments not only enable efficient maintenance and enhance safety but also support emerging applications, such as autonomous driving and intelligent transportation systems.

Conventional approaches for acquiring detailed highway information typically rely on manual in situ surveys and visual inspections. While these traditional methods can be accurate, they have several limitations, including high cost, low update frequency, and the frequent need for temporary road or lane closures to ensure safety [1,2]. The advent of Mobile Laser Scanning (MLS) technology, which integrates Light Detection and Ranging (LiDAR), Global Navigation Satellite Systems (GNSS), and Inertial Navigation Systems (INS), has enabled the collection of dense and highly accurate 3D measurements [3]. When deployed on highways, an MLS system continuously samples the environment, capturing the geometry of objects as dense three-dimensional (3D) point clouds that include rich information, such as 3D coordinates, intensity, and acquisition time. In recent years, MLS-acquired point clouds have been applied in various road-related tasks, including road surface segmentation [4,5,6,7], curbstone and marking extraction [8,9], and object detection and classification in urban road settings [10,11]. However, there remains a lack of automatic methods for detailed semantic segmentation and reconstruction of highway environments from MLS point clouds.

To address this gap, we present an automatic method for fine-grained semantic segmentation and reconstruction of highway environments using dense 3D point clouds acquired by MLS. The method first performs semantic segmentation to assign detailed semantic labels to the point cloud data, followed by reconstruction of the highway environment based on these segmentation results. The main contributions of this work are as follows:

1. A multi-scale data augmentation method is introduced to mitigate the imbalance of training samples in the original point cloud dataset.
2. A fine-grained semantic segmentation algorithm is developed, combining multi-dimensional geometric feature extraction with a KPConv convolutional network for 3D point cloud data, thereby improving overall segmentation accuracy.
3. An automatic workflow for 3D reconstruction of highway environments is proposed, utilizing the results of fine-grained semantic segmentation.

The remainder of this paper is organized as follows. Related work is reviewed in Section 2. Section 3 describes the proposed method in detail. Section 4 presents the experimental results and analysis, including quantitative and qualitative evaluations of the proposed method, comparisons with existing approaches, and a discussion of its strengths and limitations. Finally, Section 6 concludes the paper.

## 2. Related Work

This section reviews prior work on semantic segmentation and reconstruction of highway environments in Section 2.1 and Section 2.2, respectively.

### 2.1. Semantic Segmentation

Semantic segmentation classifies each point in a 3D point cloud into predefined categories based on its attributes and spatial relationships with neighboring points. The task involves assigning a semantic label to each point, effectively partitioning the point cloud into semantically meaningful object segments. Based on the employed techniques, semantic segmentation of 3D point clouds can be broadly categorized into two groups: (i) rule-based methods and (ii) learning-based methods.

#### 2.1.1. Rule-Based Methods

Traditional rule-based methods rely on hand-crafted features to classify points into different categories. A highway environment typically comprises pavements, road markings, lamp poles, road signs, and other elements. Various rule-based methods have been proposed for specific applications. Zai et al. [12] introduced a hyper-voxel generation algorithm to extract road boundaries and surfaces automatically. Wu et al. [13] presented a stepwise approach to remove off-ground points, segmenting the original point cloud vertically along the MLS trajectory and extracting ground points based on average height via RANSA [14]. C. Ma et al. [15] applied multiple thresholding dynamics according to point density at different scan distances, followed by morphological operations to segment road signs. Kim et al. [16] utilized a contour-based intensity analysis to segment road markings. Road signs were further segmented using SVM classifiers trained with histograms of oriented gradients and color descriptors [17]. Wang et al. [18] clustered raw point clouds using multi-level octrees and applied PCA to construct shape descriptors. Wang et al. [19] proposed a voxel-based region-growing method to extract pole-like objects, while Sha et al. [20] introduced a super-voxel framework to enhance road boundary delineation when the number of super-voxels is insufficient [18].

Rule-based methods require extensive expertise to design suitable features and tune parameters. Their performance is often limited by scene complexity and data quality, making fine-grained semantic segmentation challenging in complex road environments with numerous irregular objects and varying point cloud density.

#### 2.1.2. Learning-Based Methods

Learning-based methods employ neural network architectures to automatically extract features from 3D point clouds. These methods can be grouped into projection-based, voxel-based, and point-based approaches.

Projection-based methods convert point clouds into dense, regular 2D representations, either as top-down Bird’s Eye Views (BEV) [21,22] or onto spherical surfaces. Networks such as SqueezeNet are then applied to extract semantic features, with predictions refined via conditional random fields [23,24]. Similarly, range images generated from point clouds are processed with CNNs for segmentation [25]. A limitation of projection methods is the loss of spatial and structural information during 2D transformation. Lagahit et al. [26] modified Fast-SCNN with dilation blocks for improved road marking extraction, and in subsequent work [27], they proposed a focal combo loss to enhance model focus on sparse targets, achieving superior performance in accuracy and robustness.

Voxel-based methods discretize the point cloud into a voxel grid, enabling 3D CNN application. For example, VoxelNet [28] converts points into voxels for label prediction, and SVASeg [29] applies a sparse voxel-based multi-head attention mechanism to non-empty voxels. While preserving spatial structure, these approaches often suffer from high computational and memory costs.

Point-based methods process raw 3D point clouds directly [30]. Notable works include PointNet and PointNet++ [31,32], which employ point-wise MLPs and local feature pooling for semantic feature extraction. Balado et al. [33] applied PointNet to MLS data for segmenting major road structures. PointCNN [34] introduces an x-transformation for learning local neighborhoods, while KPConv [35] encodes richer neighborhood information for robust segmentation. Additional works address point density variations and class imbalance using adaptive sampling, octree-based networks, inverse feature spaces, and attention mechanisms [36,37,38,39,40,41,42].

These studies demonstrate the feasibility of deep learning for semantic segmentation of 3D point clouds. However, challenges remain for dense MLS highway data due to:

1. High geometric variability of highway objects.
2. Varying point cloud density decreasing with distance from the MLS system.
3. Severe class imbalance across categories, resulting in unequal distribution of training samples.

### 2.2. Reconstruction of Road Structure

Three-dimensional reconstruction captures key structural properties and represents them as lines, planes, or volumes with topological connectivity. Traditional approaches relied on CAD software for manual model construction, which is time-consuming, costly, and labor-intensive. Recently, automatic reconstruction methods have emerged using MLS-acquired point clouds, broadly classified as model-driven or data-driven.

Model-driven methods fit predefined geometric models to point clouds. Verma et al. [43] detected and constructed urban 3D models using parametric shapes and roof topology graphs. Ma et al. [44] and Mi et al. [45] reconstructed highway markings by detecting candidate boxes, matching shapes to geometric templates, and combining confidence scores. Model-driven methods are limited by the need for a shape library and may struggle with complex or irregular objects.

Data-driven methods reconstruct scenes by assembling primitives—lines, planes, and volumes—from the data itself. Line primitives represent linear structures, with Bézier curves commonly used for smooth curves [46]. Surface primitives such as triangular meshes model complex geometries [47,48,49]. Volumetric reconstruction can utilize plane fitting and optimization techniques [50], or algorithms like Marching Cubes [51]. Notably, Li et al. [42] linked semantic segmentation to reconstruction, generating instance masks for road markings, enabling precise vectorized representation and topological reconstruction.

Dense MLS point clouds provide accurate representations of highway environments, but robust preprocessing is necessary to achieve faithful 3D reconstruction. This work presents a comprehensive workflow to process raw MLS point clouds and reconstruct the highway environment based on fine-grained semantic segmentation results.

## 3. Methodology

**Figure 1:**
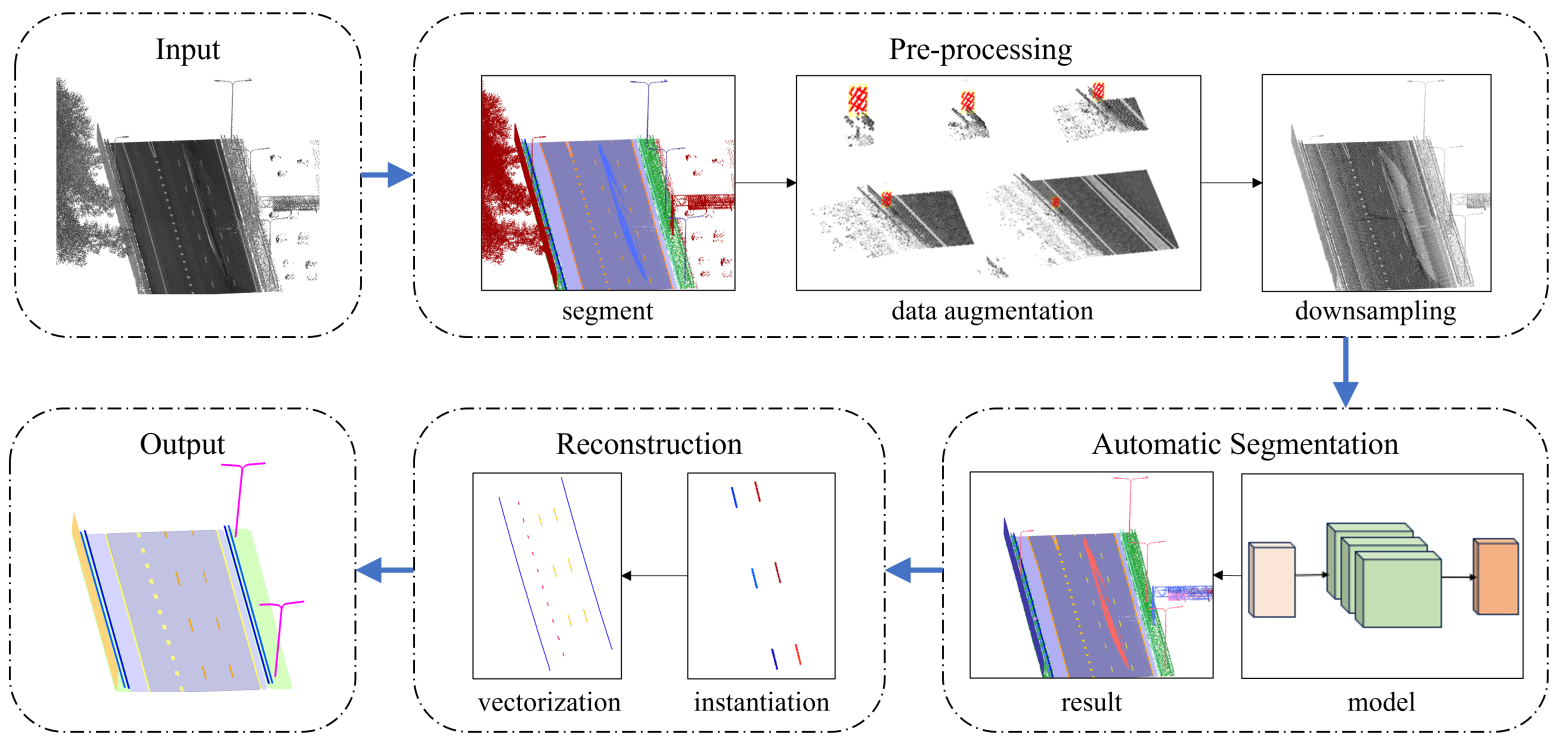
Overall workflow of the proposed methodology.

The proposed method for semantic segmentation of 3D dense point clouds acquired by MLS and the subsequent reconstruction of highway environments consists of three main steps: pre-processing, semantic segmentation, and 3D reconstruction, as illustrated in Figure 1.

### 3.1. Pre-Processing

MLS-collected point clouds are dense and voluminous, containing a wide variety of road environment objects that vary in scale and exhibit uneven distributions. To efficiently process these data, we adopt a two-step pre-processing strategy: data augmentation and down-sampling.

#### 3.1.1. Data Augmentation

The 3D point cloud data in highway environment consists of objects of varied categories, scales, and quantities. For instance, the point cloud data covering the road surface spans a wide area and exhibits high density, constituting more than half of the total volume of point cloud data. In contrast, objects such as lamp poles, posited further from the road center, are of sparse density and have less number of points. This variation in object scale and density poses challenges for the semantic segmentation process, making it difficult to accurately learn the features of these specific categories of objects, and often leads to suboptimal segmentation results. To address this issue, this work implement a multi-scale, object-based data augmentation method, aiming to enhance the quality of semantic segmentation for these particular object categories.

The multi-scale, object-based data augmentation consists of two steps:

(1) Locating objects of the categories which have fewer number of quantity: The indices of the points are used to identify 3D points representing objects of these categories, which are then clustered to form point clusters.

(2) Cropping the perimeter of the localized objects to obtain point cloud blocks: The point cloud is cropped using different sizes to produce blocks of different scales.

Smaller-scale point cloud blocks enable the model to more frequently capture the local features of objects in these categories that have fewer number of quantity. Conversely, larger-scale point cloud blocks allow the model to learn more global features. This approach selectively incorporates the cropped portions of point cloud blocks into the datasets, effectively saving memory space and improving storage efficiency, especially when dealing with large volume point cloud data.

**Figure 2:**
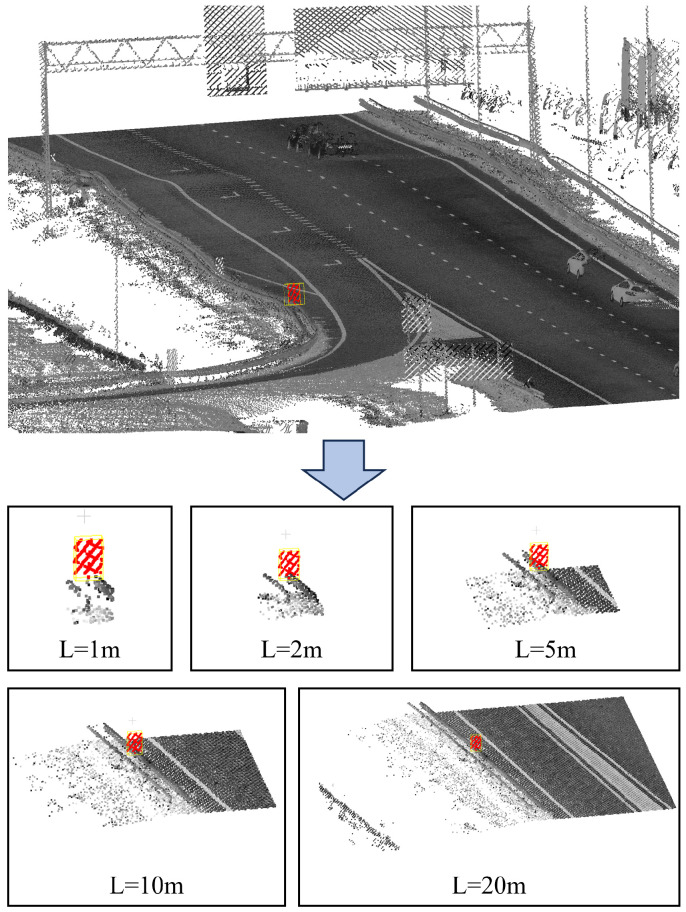
Schematicillustration of data augmentation for small traffic signs. The point cloud is color-coded by intensity, with red markers indicating small signs located on highway guardrails. Adjacent points are cropped into segmented regions with lengths of 1 m, 2 m, 5 m, 10 m, and 20 m, respectively.

Figure 2 illustrates an implementation of the data augmentation method. Point cloud in the neighborhood of the small traffic sign on the guardrail of highway are clipped out at different scales.

#### 3.1.2. Down-Sampling

The dense 3D point cloud data obtained by MLS varies in density, which areas near the scanning trajectory exhibiting a higher density, and areas further away on both sides of the trajectory having a lower density. For instance, the pavement point cloud often represents about 90% of the total road environment and features similar characteristics, leading to redundant computations during semantic segmentation. Thus, applying grid down-sampling to the input data results in a more uniform data input and mitigates the issue of unbalanced data volumes. The specific process is as follows:

(1) Calculate the bounding box and divide it into grids of a specified size;

(2) Each grid contains several points, and the point closest to the geometric center is selected as the representative sampling point.

**Figure 3:**
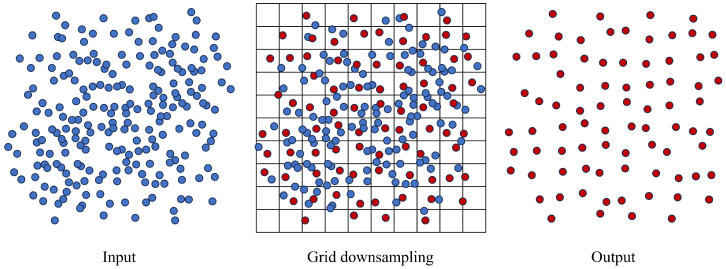
The procedure of grid-based down-sampling of the 3D dense point clouds. The points in blue color represents the original point cloud and the red color represents the sampled point cloud.

The down-sampling procedure is demonstrated in Figure 3.

### 3.2. Semantic Segmentation

In point-based semantic segmentation methods, KPConv directly processes point cloud data through point convolutions. Notably, deformable KPConv exhibits strong performance in the semantic segmentation of complex scenes. However, since its network input is limited to the x,y,z coordinates of points, its segmentation capability may become suboptimal in scenarios characterized by class imbalance or large variations in spatial scale.

**Figure 4:**
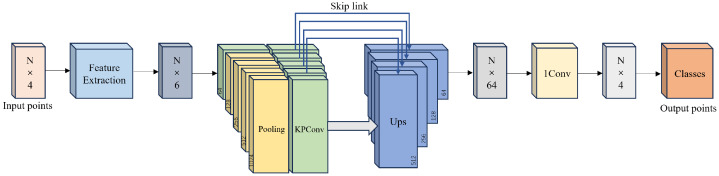
The overall procedure of the proposed semantic segmentation.

To address this, the proposed method integrates a feature extraction module with KPConv convolutional blocks to perform semantic segmentation on dense 3D point clouds. The overall workflow is illustrated in Figure 4. The feature extraction module is designed to capture local geometric characteristics from the point clouds, thereby enriching their geometric context and enhancing segmentation performance across multiple semantic categories. The details of the proposed model are described below.

#### 3.2.1. Feature Extraction Module

To enhance the local description of the point cloud for multi-category semantic segmentation, this work incorporates a feature extraction module. Different objects within the highway environment exhibit variations in shape and location distribution. For instance, elements such as traffic signs, the ground surface and protective walls exhibit distinct planar characteristics, while lamp poles, utility poles, and road side trees demonstrate pronounced verticality relative to the ground. Based on these observations, planarity *P* and verticality *V* have been selected as the geometric feature descriptors for the point cloud to enrich its local description. For each point $p_{i}$, these features are calculated based on the covariance tensor *M* using Equation (1).

(1) $$M=\frac{1}{N}\sum_{n\in P_{i}^{N}}\left(p_{n}-\bar{p}\right){\left(p_{n}-\bar{p}\right)}^{T}$$

Here, $P^{N}$ denotes the set of *N* neighboring points around $p_{i}$, and $\bar{p}$ represents their centroid. The computation of the geometric feature descriptors relies on the eigenvalues $\lambda_{1}\geq \lambda_{2}\geq \lambda_{3}\geq 0$ and the corresponding eigenvectors $e_{1},e_{2},e_{3}$ of the covariance tensor. The planarity *P* and verticality *V* are defined in Equation (2):

(2) $$\left\{P,V\right\}=\left\{\frac{\lambda_{2}-\lambda_{3}}{\lambda_{1}},1-\left|\langle \left[0\;0\;1\right],e_{3}\rangle \right|\right\}$$

**Figure 5:**
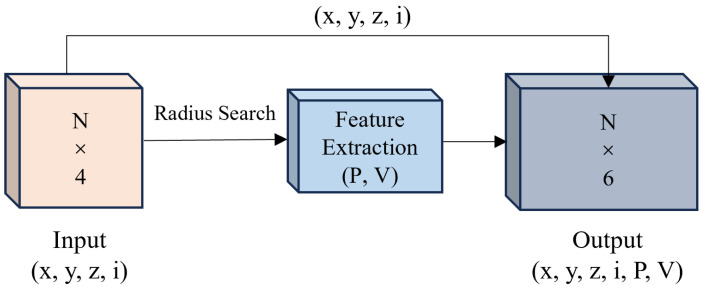
Network structure of the feature extraction module. Input data consist of 3D coordinates (x,y,z) and original intensity.

By incorporating these local geometric features, prior structural information is encoded into the model, strengthening its ability to recognize objects with diverse shapes. As illustrated in Figure 5, a radius-based neighborhood search is first conducted to retrieve points within radius *R*, followed by the computation of geometric descriptors. The resulting 3D coordinates (x,y,z), original intensity (i), and computed geometric features (P,V) are concatenated to construct a 6-dimensional feature vector for each point, denoted as p(x,y,z,i,P,V).

#### 3.2.2. Network Architecture

The semantic segmentation network follows an encoder–decoder architecture. The encoder consists of five convolutional layers, each responsible for kernel-weight computation, feature updating, and point down-sampling through max pooling. The decoder reconstructs per-point features using nearest-neighbor up-sampling. Skip connections transmit intermediate-level features from the encoder to the decoder, where they are concatenated with up-sampled features and further processed by one-dimensional convolutions to generate the final semantic labels.

Deformable KPConv is adopted as the convolution operator in this architecture. KPConv computes convolutional weights in Euclidean space based on kernel points and applies them to neighboring input points. Its ability to accommodate an arbitrary number of kernel points renders it more flexible than traditional grid-based convolutions. The deformable variant additionally learns local kernel displacements, enabling the convolutional kernels to adapt their spatial support to varying geometric regions in the input cloud. This adaptability improves robustness across diverse scenes.

**Figure 6:**
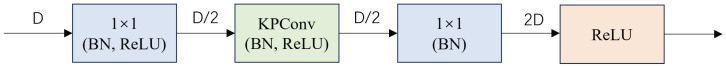
The network structure of the convolutional block. D denotes the feature dimension, taking values from the set {64, 128, 256, 512, 1024}.

The detailed structure of the deformable KPConv convolutional block is shown in Figure 6.

### 3.3. 3D Reconstruction

After obtaining the point cloud with semantic labels, the road surface and relevant roadside elements are reconstructed. The reconstruction process consists of two main stages: instance generation and vectorization.

#### 3.3.1. Instantiation

Since all points belonging to the same semantic category are assigned identical labels after semantic segmentation, an instance-level separation is required to isolate individual objects for subsequent vectorization.

In this work, the Density-Based Spatial Clustering of Applications with Noise (DBSCAN) [52] algorithm is adopted for point cloud instance segmentation. DBSCAN leverages local density characteristics and is capable of identifying clusters of arbitrary shapes. To accommodate the heterogeneous density distributions and physical dimensions of different road objects, the core parameters (ε and minPts) are configured on a per-category basis rather than using global constants. Specifically, sparse and thin objects (e.g., road markings) use ε=0.20 m, minPts = 5; dense linear objects (e.g., guardrails) use ε = 0.10 m, minPts = 8; moderately dense compact objects (e.g., lamp poles) use ε = 0.30 m, minPts = 5; and large sparse structures (e.g., billboards) use ε = 0.50 m, minPts = 10.

The execution procedure of DBSCAN can be summarized as follows:

(1) Core point selection. For each point, count the number of neighbors within its ε-neighborhood. Points with counts exceeding minPts are designated as core points.

(2) Neighborhood connectivity construction. For every core point, connect all points within its ε-neighborhood to form a temporary cluster.

(3) Cluster merging. If a point in a temporary cluster is also a core point, its corresponding temporary cluster is merged with the current one to form an expanded cluster.

(4) Noise identification. Points that are neither core points nor density-reachable from any core point are labeled as noise.

**Figure 7:**
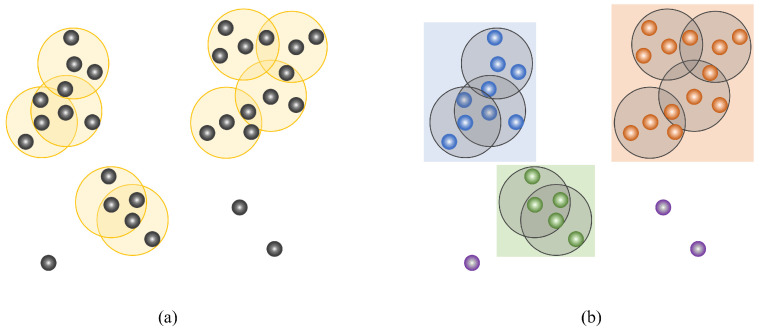
The procedure of DBSCAN clustering. (**a**) Identification of core points and creation of temporary clusters. (**b**) Merging of temporary clusters into final clusters. Yellow denotes temporary clusters, while blue, orange, and green represent the resulting clusters.

Figure 7 illustrates the DBSCAN clustering process.

#### 3.3.2. Vectorization

Point cloud vectorization is performed according to the geometric characteristics of different road objects and can be categorized into line-based and plane-based methods.

For linear objects, Bézier curve fitting is employed. A Bézier curve is defined by its endpoints and a sequence of control points, whose configuration determines the resulting curve shape. Bézier curves possess several advantages, including geometric interpretability, flexibility in control point adjustment, uniformity independent of curve parametrization, as well as invariance to translation and rotation, making them suitable for modeling linear roadside structures.

Given the control points $P_{0},P_{1},\ldots ,P_{n}$, the expression for an *n*-th order Bézier curve is as described by Equation (3):

(3) B(t)=∑i=0nniPi(1−t)n−iti

The curve passes through the start point $P_{0}$ and the end point $P_{n}$, while the intermediate control points $P_{1}∼P_{n-1}$ influence the curve shape but are not necessarily interpolated.

For planar objects, the vectorization process is carried out in the following steps:

(1) Fit a plane to the point cloud using the RANSAC algorithm.

(2) Project all points onto the fitted plane.

(3) Extract the boundary using the alpha shapes algorithm.

The overall workflow of the alpha shapes procedure is presented in Algorithm 1:
**Algorithm 1** Alpha shapes algorithm1:**Input:** Read data *p* and radius *r*2:Initialize outline as an empty array3:**for** *i* = 1 to length(*p*) **do**4:      $p_{0}$ = p[0]5:      pr = *p* (remove $p_{0}$)6:      pr_cir = pr (inner 2r)7:      **for** *j* = 1 to length(pr_cir) **do**8:            $p_{1}$ = pr_cir[0]9:            pr_cirr = pr_cir (remove $p_{1}$)10:            $p_{2}$ = $cir1(p_{0},p_{1})$11:            $p_{3}$ = $cir2(p_{0},p_{1})$12:            **for** *k* = 1 to length(pr_cirr) **do**13:                 $p_{k}=pr\_cirr\left[k\right]$14:                 $d_{1}\left[k\right]=\mathrm{distance}\left(p_{2},p_{k}\right)$15:                 $d_{2}\left[k\right]=\mathrm{distance}\left(p_{3},p_{k}\right)$16:            **end for**17:            ${\mathrm{mind}}_{1}=\min\left(d_{1}\right)$18:            ${\mathrm{mind}}_{2}=\min\left(d_{2}\right)$19:            **if** ${\mathrm{mind}}_{1}\geq r$ or ${\mathrm{mind}}_{2}\geq r$ **then**20:                 Add $p_{1}$ to outline21:                 **break**22:            **end if**23:      **end for**24:**end for**25:**Output:** Obtain outline

## 4. Experimentation and Analysis

In this work, several experiments are conducted to evaluate the proposed methodology. Section 4.1 describes the dataset and implementation used in this paper. Section 4.2 presents the experimental results for the semantic segmentation part. Section 4.3 presents the experimental results for the 3D reconstruction part.

### 4.1. Data Description and Implementation Details

#### 4.1.1. Datasets and Metrics

This work employs a highway-scale point cloud dataset acquired from a 32 km roadway segment in the Netherlands, consisting of approximately 430 million points. The dataset is fully and manually annotated to generate high-quality ground-truth labels for training and evaluation. Table 1 summarizes the statistics of all categories, including point counts per class and instance counts for discrete object types. The dataset contains 27 finely annotated categories that cover diverse highway assets, such as single-side guardrails, protective walls, dashed lane markings of different lengths, and multiple lamp pole types. The point distribution across categories is notably imbalanced: ground-related classes (Class 14 and Class 15) comprise more than 78% of all points, whereas critical yet sparse highway facilities—such as billboards and spanning road signs (Class 23, 24, and 25)—contribute less than 1%. Owing to its scale, granularity, and high degree of imbalance, this dataset is well suited for evaluating multi-class fine-grained semantic segmentation and large-scale 3D reconstruction.

**Figure 8:**
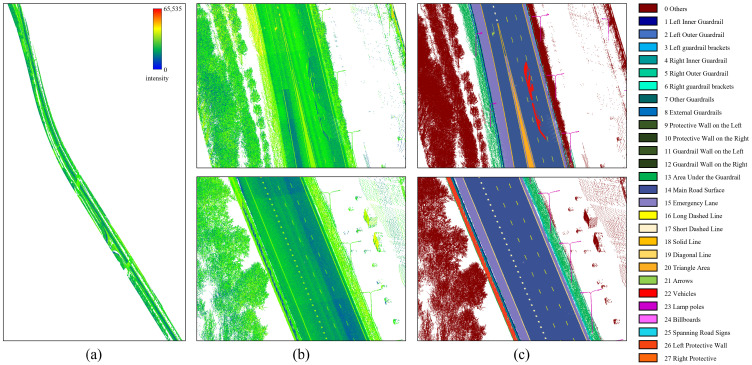
The used validation dataset in this work. (**a**) Overall road intensity information. (**b**) Raw input point cloud. (**c**) Labeled ground truth.

Figure 8 provides a visualization of the dataset. The raw point cloud is colored by intensity, ranging from blue (low intensity) to red (high intensity). All manually annotated semantic categories are displayed with distinct colors to illustrate the spatial distribution of different object classes.

During preprocessing, the entire dataset is divided into 791 non-overlapping tiles along the driving trajectory, each covering 40 m in length. Among these tiles, 100 are used for validation, 100 for testing, and the remaining tiles form the training set.

To evaluate both semantic segmentation and geometric reconstruction, we employ a set of quantitative metrics that jointly assess classification accuracy and geometric fidelity. For semantic segmentation, the primary indicator is the Mean Intersection over Union (mIoU), defined as:

(4) $$\mathrm{mIoU}=\frac{1}{k}\sum_{i=1}^{k}\frac{TP_{i}}{TP_{i}+FP_{i}+FN_{i}},$$

where $TP_{i}$, $FP_{i}$, and $FN_{i}$ represent the true positives, false positives, and false negatives of class *i*, respectively, and *k* denotes the total number of semantic classes. To complement mIoU and provide a more detailed characterization of segmentation quality, we further report the overall accuracy, which measures the proportion of correctly classified points:

(5) $$\mathrm{Accuracy}=\frac{TP+TN}{TP+TN+FP+FN},$$

as well as the per-class recall, which reflects the completeness of segmentation for each category:

(6) $${\mathrm{Recall}}_{i}=\frac{TP_{i}}{TP_{i}+FN_{i}}.$$

For geometric reconstruction, fidelity to the underlying structure is assessed using distance-based metrics. The Root Mean Square Error (RMSE) quantifies the average squared deviation between points and their fitted geometric representation:

(7) $$\mathrm{RMSE}=\sqrt{\frac{1}{N}\sum_{i=1}^{N}d_{i}^{2}},$$

while the Mean Distance (MD) is used to characterize the average absolute deviation of points from the model:

(8) $$\mathrm{MD}=\frac{1}{N}\sum_{i=1}^{N}\left|d_{i}\right|,$$

where $d_{i}$ denotes the Euclidean distance between the *i*-th point and its corresponding geometric representation, and *N* is the total number of points evaluated. These complementary metrics provide a comprehensive assessment framework, capturing both the geometric accuracy of the reconstructed vectorized structures and, when combined with semantic metrics, the overall quality of multi-class segmentation.

#### 4.1.2. Implementation Details

All experiments are conducted on a 64-bit Linux Ubuntu environment. Table 2 summarizes the hardware and software configuration, and Table 3 lists the parameters used in our experiments. These parameter settings are determined through empirical evaluation, and detailed parameter analyses are provided in Section 4.2.3.

### 4.2. Segmentation Results

#### 4.2.1. Comparison to State-of-the-Art Methods

To validate the effectiveness of the proposed multi-class semantic segmentation method for highway scenarios, we compared it with several representative state-of-the-art methods, including PointNet++ [32], DGCNN [53], and KPConv [35]. The segmentation accuracy results are summarized in Table 4, which includes both overall metrics—mean Intersection over Union (mIoU), overall accuracy (OA), and mean recall—and per-class performance. As shown, the proposed method achieves excellent segmentation results across all 27 classes, with overall performance reaching 90.20% OA and 82.27% mIoU, effectively addressing common challenges in fine-grained semantic segmentation for specific categories.

Compared to PointNet++, DGCNN, and KPConv, the proposed method significantly improves the recognition of small-sized and feature-similar targets, with mIoU gains of approximately 18–42%. This improvement is mainly attributed to the multi-scale data augmentation strategy and the geometric feature extraction module, which enhance the model’s ability to learn underrepresented small categories such as External Guardrails, Arrows, Lamp Poles, and Billboards, resulting in notable accuracy gains (compared with KPConv, improvements are approximately 9%, 67%, 1%, and 30%, respectively). In contrast, methods such as KPConv and DGCNN, which rely solely on sampled convolution features, exhibit limited capability in capturing discriminative features for small and low- abundance classes.

**Figure 9:**
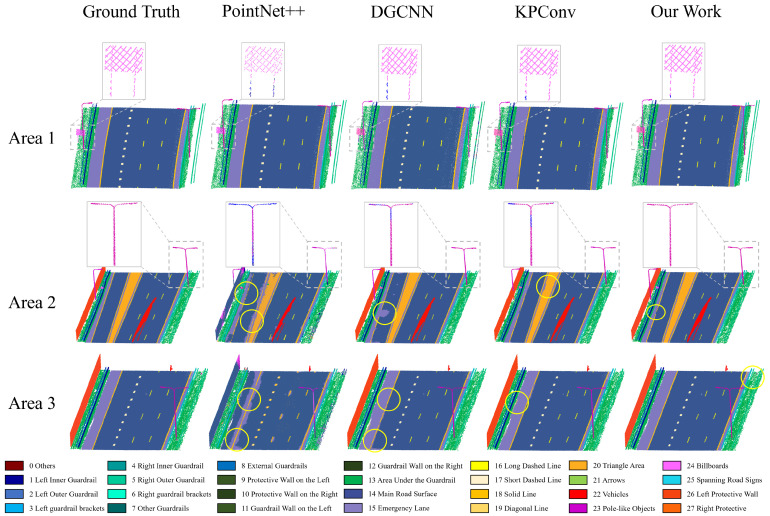
Comparison of semantic segmentation results of different methods. The grey boxes and connected interest maps show some of the detail in the road, and the yellow circles mark areas where there are errors in segmentation.

Figure 9 presents the visualization results of semantic segmentation across different methods, covering diverse road scenarios and partial category targets. As illustrated, the proposed method consistently outperforms other approaches in various segmentation tasks. For instance, in the case of billboards, whose sizes and shapes vary, some regions are easily misclassified as surrounding categories. The geometric feature module in our approach effectively models such geometrically distinct objects, thereby substantially improving segmentation accuracy for these targets.

**Figure 10:**
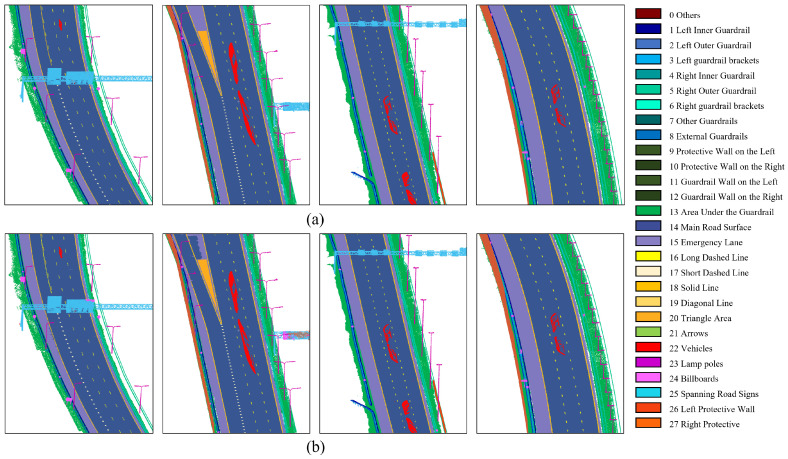
Semantic segmentation results for different road sections. (**a**) Ground truth. (**b**) Results of our work.

Figure 10 illustrates the overall semantic segmentation results obtained by the proposed method. Figure 10a shows the ground truth annotations for different road segments, while Figure 10b presents the corresponding segmentation outputs. The results demonstrate that our method can effectively achieve multi-class semantic understanding in highway scenarios, supporting applications such as asset inventory and large-scale 3D reconstruction. Furthermore, these accurate semantic outputs provide a reliable foundation for the subsequent geometric reconstruction of highway infrastructure.

#### 4.2.2. Ablation Study

To validate the effectiveness of the proposed key components (geometric module and multi-scale data augmentation) and their incremental contributions to fine-grained segmentation, we conduct systematic ablation experiments. The original KPConv is adopted as the baseline, and all model variants are trained under identical configurations (optimizer, learning rate, training epochs, etc.) to ensure fair comparison. The evaluation focuses on overall mIoU and minority class mIoU specifically targeting three typical fine-grained categories (External Guardrails, Arrows, and Billboards) to highlight the improvements in challenging minority class segmentation.

As presented in Table 5, the performance degradation of model variants after removing key components directly reflects their contribution to both overall and minority class segmentation. When the geometric module is removed, the overall mIoU drops by 5.34% compared to the full proposed model, while the minority class mIoU (External Guardrails/Arrows/Billboards) declines by 12.44%. This significant drop confirms that the geometric module enables the model to learn discriminative spatial geometric features—such as the slender structure of guardrails, the sparse point distribution of arrows, and the irregular surface contours of billboards—which is crucial for resolving ambiguities in fine-grained minority class segmentation that the baseline KPConv cannot address. Such targeted feature learning aligns with the key insight that geometric cues are critical for distinguishing similar or low-data categories in point cloud segmentation.

Removing the multi-scale data augmentation leads to a 3.62% decrease in overall mIoU and an 8.57% drop in minority class mIoU. This indicates that multi-scale data augmentation effectively enriches the diversity of training samples for underrepresented categories, reducing the model’s bias toward majority classes and enhancing its generalization ability to variable scales of guardrails, arrows, and billboards in complex road scenarios. The greater impact on minority classes is consistent with the value of data augmentation in compensating for limited annotated samples of fine-grained categories.

Comparing all model variants, the full proposed model achieves the best performance across all metrics. It outperforms the baseline KPConv by 17.32% in overall mIoU and by 35.01% in minority class mIoU, highlighting the significant technical advantage of the proposed method. This validates that the geometric module and multi-scale data augmentation are indispensable and synergistic: the geometric module provides the capability to capture fine-grained geometric features of minority classes, while data augmentation ensures the model learns these features from diverse samples. Their combination constitutes the key technical contribution, effectively enhancing the segmentation performance of challenging minority categories beyond the baseline’s capabilities.

#### 4.2.3. Parameter Analysis

To justify the selection of key parameters (neighborhood radius and grid sampling size), we conducted a sensitivity study by evaluating their impact on model performance. As shown in Table 6, the neighborhood radius of 1.8m was chosen because it balances the capture of local geometric context (critical for distinguishing fine-grained objects like arrows and guardrails) and computational efficiency: smaller radii (≤1.2 m) fail to include adjacent structural features, while larger radii (>2.4 m) introduce redundant background information. For grid sampling size, 0.04m optimally preserves details of small objects (e.g., thin guardrails and billboard edges) without excessive memory usage—coarser sampling (≥0.06 m) leads to information loss, while finer sampling (≤0.02 m) increases computational cost without significant performance gains.

### 4.3. Reconstruction Results

The goal of geometric reconstruction in highway scenarios is to perform instance-level modeling, key feature point extraction, and vectorization of objects based on the preceding multi-class fine-grained semantic segmentation. These processes enable accurate 3D reconstruction of roads and associated infrastructure, providing a structured representation of both the roadway and its assets.

#### 4.3.1. Instantiation Results

**Figure 11:**
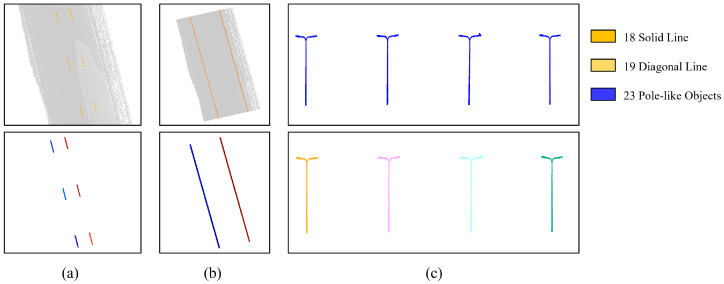
Results of instantiated segmentation. (**a**) Long Dashed Line. (**b**) Solid Line. (**c**) Lamp poles. Below, different instances after segmentation are displayed with random colors.

The point cloud corresponding to elements of interest is first extracted and instantiated using DBSCAN clustering with category-specific parameters: sparse road markings (e.g., dashed lines) use ε=0.20 m and minPts=5, moderately dense lamp poles use ε=0.30 m and minPts=5, and dense guardrails use ε=0.10 m and minPts=8, tailored to their respective point cloud density and physical size. This process segments the point cloud into distinct entities, such as a single lane line, a single guardrail, and a single lamp pole, among others. Specifically for the dashed line in the middle of the road, each small segment requires separate fitting. The results of this instantiation and segmentation are illustrated in Figure 11.

#### 4.3.2. Vectorization Results

After segmenting each category into individual point clouds, keypoints are extracted and used for reconstruction. For point clouds with pronounced linear features, such as lane lines and guardrails, the segmentation is initially performed along the direction of the principal components. Subsequently, the center point of each segmented point cloud is calculated to approximate the trajectory along the center line. In the experiment, blocks were divided at intervals of 0.4m. On average, each short segment of the long dashed line in the middle of the road comprised 7 points. In the 40m-long point cloud blocks, the borderline and guardrail on both sides of the road each had an average of 100 points.

**Figure 12:**
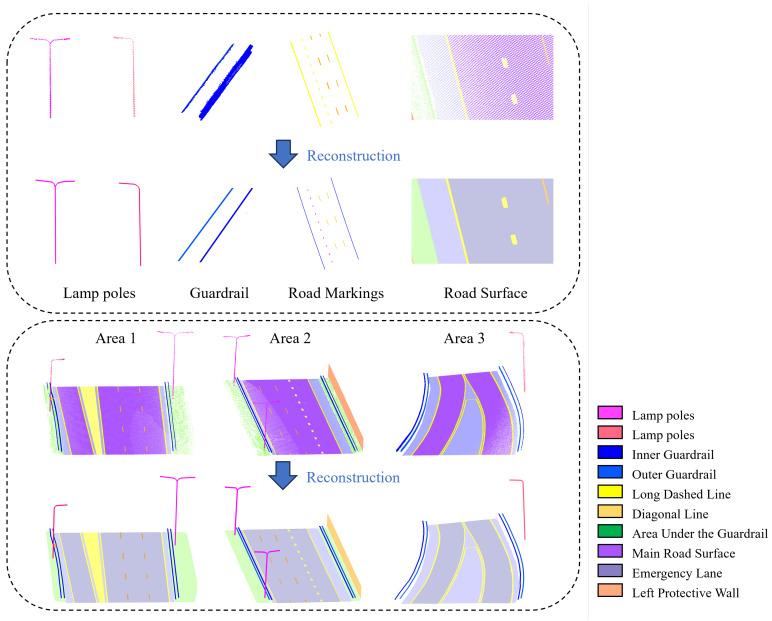
Results of reconstruction of lamp poles, guardrails, road markings, road surface and the overall view.

For linear elements, the computed center point serves as the control point and is fitted using an N-order Bézier curve. For lamp poles, key structural points are extracted and selectively fitted using straight lines. For objects such as pavements, billboards, and protective walls, planar fitting is applied. The experimental results are depicted in Figure 12.

To further validate the geometric accuracy of the vectorization, Table 7 presents the quantitative evaluation results for representative categories using RMSE and mean distance.

As shown in Table 7, all categories achieve low reconstruction errors: linear objects (guardrails, arrows, lamp poles) have RMSE below 0.032 m, with road markings (arrows) showing the highest precision (RMSE = 0.018 m) due to their regular shape. Planar objects (road surface, billboards) exhibit slightly higher errors (RMSE < 0.045 m), which is attributed to minor surface irregularities in the original point clouds. These results confirm that the proposed vectorization method maintains high geometric fidelity, with errors well within the acceptable range for practical applications (e.g., road maintenance and digital mapping).

## 5. Discussion

The proposed framework integrates multi-scale data augmentation, fine-grained semantic segmentation, and geometry-aware vectorization techniques to achieve automated reconstruction of highway environments from dense mobile laser scanning (MLS) point clouds. The method effectively addresses key challenges such as class imbalance, multi-category segmentation, and small-object detection. Its effectiveness is validated through extensive experiments, and the performance, robustness, and potential limitations are further analyzed.

In the fine-grained semantic segmentation module, the combination of multi-dimensional geometric features with a KPConv backbone substantially enhances the recognition of small and underrepresented categories. The model achieves consistently high accuracy across most target classes, with particularly strong performance on linear road elements such as lane markings, road edges, and guardrails. Nonetheless, the method remains dependent on high-quality annotated data, and its robustness under noisy or low-sample conditions still leaves room for improvement. Meanwhile, although the multi-scale augmentation strategy provides a simple and effective sample-level solution for mitigating class imbalance, it does not fully resolve the challenge of distinguishing visually similar categories, as highlighted in prior work [39,40]. Future efforts will focus on improving discrimination among semantically and geometrically similar classes to further advance fine-grained segmentation.

In the geometric reconstruction stage, DBSCAN-based instance segmentation demonstrates strong performance on structurally coherent objects. With class-specific parameterization, the method adapts well to varying density patterns—from sparse dashed markings to dense guardrail structures—and produces reasonable clustering results. However, the inherent density sensitivity of DBSCAN can cause fragmentation in regions with abrupt density changes (e.g., occlusions or distant roadside objects), necessitating additional post-processing or structural priors for refinement. The subsequent vectorization step achieves high geometric fidelity: linear elements are effectively represented using Bézier curve fitting, yielding smooth and consistent representations even under irregular point sampling; planar elements are robustly modeled using RANSAC-based fitting, with reconstruction errors primarily attributable to natural road surface roughness and sensor noise. These errors remain well within acceptable limits for practical applications such as road maintenance, asset inventory, and digital-twin construction.

Overall, the proposed framework demonstrates strong performance in both fine-grained semantic segmentation and geometric reconstruction, underscoring its potential in highway asset digitization and intelligent transportation applications. Future research will explore learning-based instance segmentation mechanisms and more adaptive vectorization strategies to better accommodate local geometric variability and complex topological structures, ultimately moving toward a fully end-to-end reconstruction pipeline.

## 6. Conclusions

This work presents an integrated framework for fine-grained semantic segmentation and geometric reconstruction of highway environments based on dense mobile laser scanning (MLS) point clouds. The proposed approach combines multi-scale data augmentation, geometry-aware feature extraction, and instance-level vectorization to enable accurate recognition and modeling of linear and planar roadway infrastructure elements.

Experimental results demonstrate that the framework achieves state-of-the-art performance across 27 highway-related categories, substantially improving the recognition of small, sparse, or visually similar objects such as external guardrails, arrow markings, lamp poles, and billboards. Ablation studies further validate the critical contributions of the geometric feature module and multi-scale data augmentation, particularly for minority classes and challenging segmentation tasks. The geometric reconstruction module successfully instantiates segmented objects and generates high-fidelity vector representations. Linear elements are effectively modeled using Bézier curves, while planar elements are robustly fitted using RANSAC-based plane fitting. Quantitative evaluation shows that reconstruction errors for all representative categories remain low, confirming that the proposed method preserves geometric accuracy of the components and is suitable for practical applications including asset inventory, road maintenance, and digital twin construction.

In summary, the proposed framework provides a reliable and efficient solution for multi-class semantic understanding and high-precision geometric reconstruction of highway environments, offering valuable technical support for intelligent transportation systems and digital infrastructure management.

## Figures and Tables

**Table 1:** Proportion of point counts and the number of instances for each category in the dataset. The hyphen “–” in the Instance Count column indicates non-discrete categories for which instance statistics are not applicable.

Class Label	Class Name	Point Count	Percentage (%)	Instance Count
0	Others	964,283	0.22	–
1	Left Inner Guardrail	5,655,092	1.32	–
2	Left Outer Guardrail	2,385,949	0.56	–
3	Left Guardrail Brackets	573,165	0.13	–
4	Right Inner Guardrail	3,663,719	0.86	–
5	Right Outer Guardrail	1,067,070	0.25	–
6	Right Guardrail Brackets	301,406	0.07	–
7	Other Guardrails	3,082,817	0.72	–
8	External Guardrails	116,136	0.03	–
9	Protective Wall on the Left	721,971	0.17	–
10	Protective Wall on the Right	1,907,323	0.45	–
11	Guardrail Wall on the Left	704,418	0.16	–
12	Guardrail Wall on the Right	1,084,853	0.25	–
13	Area Under the Guardrail	17,164,924	4.01	–
14	Main Road Surface	283,676,569	66.26	–
15	Emergency Lane	53,061,488	12.39	–
16	Long Dashed Line	1,938,922	0.45	5194
17	Short Dashed Line	1,200,440	0.28	2456
18	Solid Line	9,134,953	2.13	2285
19	Diagonal Line	361,395	0.08	126
20	Triangle Area	1,935,071	0.45	85
21	Arrows	272,262	0.06	187
22	Vehicles	3,881,077	0.91	517
23	Lamp Poles	1,011,025	0.24	1727
24	Billboards	621,812	0.15	829
25	Spanning Road Signs	2,209,391	0.52	123
26	Left Protective Wall	24,052,947	5.62	–
27	Right Protective	5,383,467	1.26	–

**Table 2:** Specifications of the experimental environment.

Experimental Environment	Configuration
Operating System	Ubuntu20.04
CPU	Inter RXeon(R) Silver 4210R CPU@2.40 GHz × 40
GPU	NVIDIA Corporation GV100[TITAN V]
RAM	64 GB
VRAM	12 GB
Deep Learning Framework	PyTorch 2.1
Python	Python 3.10

**Table 3:** Parameter Settings.

Parameter	Value
Optimizer	Momentum Gradient Descent
Loss Function	Cross-Entropy
Batch Size	10
Momentum	0.98
Learning Rate	0.01
Max Training epoch	500
Neighborhood Radius for Feature Extraction	1.8 m
Grid Sampling Size	0.04 m

**Table 4:** Comparison of semantic segmentation results across different methods. The bold black text indicates the best value in each row.

Class Label	Class Name	PointNet++	DGCNN	KPConv	Our Work
	mIoU	44.98	57.55	69.95	**87.27**
	Accuracy	62.35	71.82	78.30	**90.20**
	Mean Recall	48.67	56.43	65.20	**82.60**
1	Left Inner Guardrail	73.52	84.38	91.30	**95.10**
2	Left Outer Guardrail	45.08	53.75	**91.81**	90.87
3	Left Guardrail Brackets	35.2	39.32	58.85	**58.98**
4	Right Inner Guardrail	87.54	87.9	93.14	**99.53**
5	Right Outer Guardrail	54.41	43.34	91.16	**96.36**
6	Right Guardrail Brackets	8.31	35.92	54.40	**62.6**
7	Other Guardrails	56.75	72.18	97.36	**98.73**
8	External Guardrails	0	0	77.78	**86.11**
9	Protective Wall on the Left	21.8	84.15	69.95	**87.3**
10	Protective Wall on the Right	18.97	72.65	20.17	**99.93**
11	Guardrail Wall on the Left	25.02	47.46	64.28	**95.80**
12	Guardrail Wall on the Right	50.13	37.22	31.53	**88.24**
13	Area Under the Guardrail	77.42	93.35	96.96	**97.19**
14	Main Road Surface	79.81	88.23	95.13	**99.05**
15	Emergency Lane	46.6	43.09	79.89	**95.63**
16	Long Dashed Line	61.16	62.87	74.30	**81.65**
17	Short Dashed Line	21.81	24.52	81.50	**90.43**
18	Solid Line	63.45	77.65	78.58	**89.32**
19	Diagonal Line	38.17	10.29	6.39	**82.71**
20	Triangle Area	50	57.88	86.52	**95.88**
21	Arrows	0	0	0	**67.40**
22	Vehicles	91.9	**99.75**	98.27	99.17
23	Lamp Poles	40.58	39.38	90.50	**91.69**
24	Billboards	17.18	24.18	11.46	**40.76**
25	Spanning Road Signs	56.73	**98.08**	60.68	73.84
26	Left Protective Wall	65.41	93.35	96.90	**99.14**
27	Right Protective	27.53	82.86	89.72	**92.98**

**Table 5:** Ablation Results of Key Components. “w/o” denotes “without”, “Data Augmentation” specifically refers to multi-scale data augmentation. Minority class mIoU is the average of External Guardrails, Arrows, and Billboards. The bolded values represent the best performance in each column.

Model Variant	Overall mIoU (%)	Minority Class mIoU (%) (External Guardrails/Arrows/Billboards)
Proposed Model (Full)	**87.27**	**64.75**
Proposed Model (w/o Geometric Module)	81.93	52.31
Proposed Model (w/o Data Augmentation)	83.65	56.18
KPConv (Baseline)	69.95	29.74

**Table 6:** Sensitivity Analysis of Key Parameters. The values in bold black type denote the highest performance achieved under different parameters.

Parameter	Value	mIoU (%)	Accuracy (%)
Neighborhood Radius	1.2 m	82.3	86.5
	1.8 m	**87.3**	**90.2**
	2.4 m	85.1	88.7
	3.0 m	83.5	87.1
Grid Sampling Size	0.02 m	86.9	89.8
	0.04 m	**87.3**	**90.2**
	0.06 m	85.4	88.5
	0.08 m	82.8	86.3

**Table 7:** Geometric Reconstruction Errors of Vectorization for Representative Categories.

Object Category	Vectorization Method	RMSE (m)	Mean Distance (m)
Guardrails (Linear)	3rd-order Bézier Curve	0.032	0.025
Road Markings (Arrows)	2nd-order Bézier Curve	0.018	0.015
Lamp Poles (Linear)	Straight Line Fitting	0.021	0.017
Road Surface (Planar)	RANSAC Plane Fitting	0.045	0.038
Billboards (Planar)	RANSAC Plane Fitting	0.037	0.029

## Data Availability

Data are contained within the article.
